# Using High Performance Thin Layer Chromatography-Densitometry to Study the Influence of the Prion [*RNQ*^+^] and Its Determinant Prion Protein Rnq1 on Yeast Lipid Profiles

**DOI:** 10.3390/separations5010006

**Published:** 2018-01-16

**Authors:** Quang Bui, Joseph Sherma, Justin K. Hines

**Affiliations:** Department of Chemistry, Lafayette College, Easton, PA 18042, USA

**Keywords:** yeast, prion, lipid, thin layer chromatography, Rnq1, amyloid, cholesterol, phospholipid, squalene, triglyceride

## Abstract

The baker’s yeast *Saccharomyces cerevisiae* harbors multiple prions that allow for the creation of heterogeneity within otherwise clonal cell populations. However, in many cases, the consequences of prion infection are entirely unclear. Predictions of prion-induced changes in cell physiology are complicated by pleotropic effects, and detection is often limited to relatively insensitive cell growth assays that may obscure many physiological changes. We previously showed that silica gel high performance thin-layer chromatography-densitometry (HPTLC) can be used to empirically determine prion-induced changes in lipid content in yeast. Here, we conduct pair-wise quantifications of the relative levels of free sterols, free fatty acids, and triacylglycerols [petroleum ether-diethyl ether-glacial acetic acid (80:20:1, *v*/*v*/*v*) mobile phase and phosphomolybdic acid (PMA) detection reagent]; steryl esters, methyl esters, and squalene [hexane-petroleum ether-diethyl ether-glacial acetic acid (50:20:5:1, *v*/*v*/*v*/*v*) and PMA]; and phosphatidylethanolamine, phosphatidylcholine, and phosphatidylinositol (chloroform-diethyl ether-acetic acid (65:25:4.5, *v*/*v*/*v*) and cupric sulfate-phosphoric acid) in otherwise clonal prion-infected ([*RNQ*^+^]) and prion-free ([*rnq*^−^]) cells in both stationary- and logarithmic-growth phases. We detected multiple statistically significant differences between prion-infected and prion-free cells that varied by growth phase, confirming our pr evious observations that prions exert distinct influences on cell physiology between stationary- and log-phase growth. We also found significant differences between cells expressing or lacking the Rnq1 protein which forms the [*RNQ*^+^] prion, providing new clues to the as yet unresolved normal biological function of this prion-forming protein. This investigation further emphasizes the utility of HPTLC-densitometry to empirically determine the effects of prions and other presumed innocuous gene deletions on lipid content in yeast, and we expect that additional analyses will continue to resolve the physiological effects of prion infection.

## 1. Introduction

To properly function, proteins need to be folded in the correct conformation; prions are defined as misfolded forms of proteins that can convert additional normal proteins into this altered form [1]. Prions are also infectious, being able to self-propagate within a cell and, in the case of microorganisms, from mother cell to daughter cells. Many human neurodegenerative diseases have been linked with either prions or prion-like behavior of proteins, such as Alzheimer’s, Parkinson’s, Huntington’s, and mad cow disease [2,3]. Formation of a prion, however, is not a unique phenomenon in mammals. Proteins with prion-like behaviors are also being characterized in other groups of organisms, including filamentous fungi [4], sea slugs [5], plants [6], and most notably baker’s yeast [7]. Starting with the identification of [*URE3*] and [*PSI*^+^] (prion forms of proteins Ure2 and Sup35, respectively) [7], nearly a dozen prions have now been identified in the baker’s yeast *Saccharomyces cerevisiae* [8].

Incorporation into amyloid aggregates usually results in a loss of normal prion protein function, typically manifesting in a loss-of-function phenotype in the corresponding metabolic or signaling pathway and in some cases leading to cell death [1]. Prions can also interact with one another [9]. The prion [*RNQ*^+^] (sometimes called [*PIN*^+^]) is the prion form of Rnq1 protein [10] and has the unusual ability to enhance the spontaneous appearance of other prions [11]. Interestingly, deletion of the *RNQ1* gene resulted in a strain with normal growth rates, mating ability, and sporulation efficiency and initially no detectable phenotypes beyond the inability to form [*RNQ*^+^] [11]. A later study found that deletion of *RNQ1* reduced the expression of a microtubule-associated protein, Bik1 [12], which, when deleted itself, negatively affects chromosome migration and stability [13,14]. Despite this singular observation, a biological function for the Rnq1 protein is unclear. Likewise, until recently, the prion [*RNQ*^+^] has no known phenotype beyond a single instance where it enhances the suppression of a nonsense mutation in the presence of a second prion [15] and has the more dramatic ability to enhance of the formation of other prions and amyloids [10,11,16]. However, despite a lack of clear biological function, and like other prions, [*RNQ*^+^] is often found naturally in wild yeast, suggesting that it might have some unknown beneficial functionality in yeast [17]. Therefore, Rnq1, in both its prion and non-prion forms, may play a role in regulating or altering some other biological functions in *S. cerevisiae*.

Due to the potential vast complexity of the pleotropic effects that may occur, it is very difficult to predict, de novo, the physiological changes associated with prion infection. On the other hand, it is much easier to detect physiological changes empirically. Since lipids serve in a wide variety of functions in yeast cells (structural, energy storage, and signaling), in the past, we predicted that it might be likely that a change in lipid profile could be observed when a prion is present in *S. cerevisiae* [18]. A set of efficient lipid extraction procedures for yeast cells was previously described by Schneiter and Daum [19], and several analytical methods have been utilized for quantification of lipids in yeast cells, most notably high performance liquid chromatography [20] and gas chromatography-mass spectrometry [21]. In a previous study done by Schmidt et al. [22], the lipid content of lipid droplets in yeast was analyzed using only qualitative one-dimensional thin-layer chromatography (TLC) with double development for nonpolar lipids and qualitative two-dimensional TLC for phospholipids, showing the major lipid classes in yeast to be free sterol (FS), free fatty acid (FFA), triacylglycerol (TAG), and steryl ester (SE) and phosphatidylinositol (PI), phosphatidylethanolamine (PE), and phosphatidylcholine (PC), respectively. HPTLC (high performance TLC) has been utilized extensively in lipid quantification in biological samples (reviewed by Fried [23] and Bui et al. [24]). Recently, we showed that HPTLC was a robust method of quantifying lipids in yeast cells, allowing highly reproducible and accurate measurements [18].

Previously, we investigated the differences in lipid profiles of prion-free yeast cells ([*psi*^−^] and [*ure-o*]) against their respective clonal prion-infected strains ([*PSI*^+^] and [*URE3*]) in both logarithmic phase and stationary phase [18]. The presence of these prions was found to exert significant changes to lipid profiles of yeast cells in both growth phases. In addition, we also saw that the influence of prions on the yeast lipid profile varied between the growth phases being tested [18]. However, the interpretation of these results was complicated because the [*URE3*] yeast strain used also harbored the [*RNQ*^+^] prion. Thus, the degree to which changes in lipid profiles were attributable to either prion remains unclear. To address this problem, here we investigated the lipid profile of yeast cells from three different strains: a wild-type strain lacking the [*RNQ*^+^] prion (denoted [*rnq*^−^]); an otherwise identical strain possessing the [*RNQ*^+^] prion; and, finally, an otherwise identical strain lacking the *RNQ1* gene (Δ*rnq1*) and, therefore, lacking the prion-forming Rnq1 protein. Direct comparison between the [*RNQ*^+^] strain vs. the wild-type [*rnq*^−^] strain would be expected to reveal any effects on lipid profile due solely to the presence of [*RNQ*^+^] aggregates, while comparison of the Δ*rnq1* strain and the wild-type [*rnq*^−^] strain should reveal effects due to the loss of the normal function of the Rnq1 protein itself. Prior to this investigation no phenotypes related to lipids have been observed either for the presence of [*RNQ*^+^] or due to the deletion of *RNQ1*, so this investigation also tested the hypothesis that the observed changes found in Bui et al. [18] in the [*URE3*]/[*RNQ*^+^] strain were due solely to the presence of [*URE3*]. This hypothesis was nullified; our results indicated significant changes in lipid content between strains possessing or lacking [*RNQ*^+^]. Further significant differences also existed between strains expressing or lacking the Rnq1 protein. These results may provide new clues towards the determination of additional functional roles for both functional Rnq1 and its intracellular amyloid aggregates, [*RNQ*^+^] beyond prion seeding.

## 2. Materials and Methods

### 2.1. Yeast Culture and Lipid Extraction

*S. cerevisiae* W303 cells used in this study were derived from PJ513a (EACY639): [*RNQ*^+^] [*psi*^−^] [*ure-o*] *MAT a trp1-1 ura3-1 leu2-3,112 his3-11,15 ade2-1 can1-100 GAL2 met2-1 lys2-2* [25]. This strain was denoted [*RNQ*^+^] in this study. A genetically identical [*rnq*^−^] strain was obtained by curing of the [*RNQ*^+^] prion by growth on media containing 4 mM GdnHCl (EACY1974). An otherwise genetically identical strain lacking the *RNQ1* gene and denoted Δ*rnq1* herein (Y1710) was additionally gifted from the Craig lab at University of Wisconsin-Madison. All cultures (*n* = 4 for each group) were grown in yeast extract-peptone-dextrose-adenine (YPDA) medium. The medium was made by dissolving 10 g of yeast extract (No. BP9727, Fisher Scientific, Waltham, MA, USA) 20 g of peptone (Fisher Scientific), and 20 g of D-glucose (No. AC41095, Acros Organics, part of Fisher Scientific) in 1 L of deionized (DI) water. An additional 80 mg of adenine (No. A8626, Sigma-Aldrich, St. Louis, MO, USA) was dissolved after sterilization of the medium by autoclaving.

For logarithmic phase cultures, cells were grown continuously for at least six generations by subculturing in YPDA medium on a shaker (200 rpm) at 30 °C. Cells were collected when their optical density (OD), measured at 600 nm using a Genesys 20 spectrophotometer (Thermo Scientific, Waltham, MA, USA), reached 0.8. For stationary phase cultures, cells were grown overnight for at least 18 h without subculturing in liquid YPDA medium with constant agitation (200 rpm) at 30 °C. Stationary phase samples were collected when cell density reached OD of approximately 7.5. For each culture, a total of approximately 120 OD units of cells were collected by centrifuging the medium at 3300 rpm for 3 min using a bench-top centrifuge (Model 228, Fisher Scientific). The pellet was washed with DI water and centrifuged again. The wet cell weight of each pellet was recorded as sample weight. Cell pellets were then flash-frozen in liquid nitrogen and stored at −20 °C until extraction.

Lipid extraction procedures were adapted from a previous study by Schneiter and Daum [19]. *S. cerevisiae* cells were lysed by bead-beating. Each sample was separated into four 2 mL centrifuge tubes and mixed with 500 μL of methanol and approximately 0.5 g of silica beads (No. 11079105, Biospec Products, Bartlesville, OK, USA). The tubes were then disrupted using a cell disruptor (Scientific Industries, Inc., Bohemia, NY, USA) for 30 s followed by 30 s rest periods, repeated for a total of 5 min. Four tubes of the same sample were then mixed in a 250 mL beaker with 8 mL of methanol and 20 mL of chloroform. The mixture was magnetically stirred at room temperature for 1 h. Lysate was filtered using a sintered glass funnel (No. CLS3606060M, Sigma-Aldrich) and washed twice in a 125 mL separatory funnel with Folch solution (0.88% KCl). The volume of Folch solution added was approximately 25% of the chloroform-methanol (2:1, *v*/*v*) volume. The lipophilic layer was collected and evaporated. The lipid film was then dissolved with 6 mL of chloroform-methanol (2:1, *v*/*v*) and collected in Teflon lined vials (No. 03-339-22D, Fisher Scientific). The solvent was evaporated just to dryness in a warm water bath (approximately 40 °C) with a gentle flow of N_2_ gas. Samples were then reconstituted in 1.00 mL of chloroform-methanol (2:1, *v*/*v*).

### 2.2. Lipid HPTLC Quantification

All solvents and chemicals used throughout the study were analytical reagent grade purchased from Sigma-Aldrich. The standard for phospholipid analysis was Polar Lipid Mixture No. 1127 (Matreya, State College, PA, USA) containing 25% each of the following compounds: cholesterol, PE, PC (lecithin), and lysolecithin with a total of 25.0 mg in 1.00 mL of chloroform. The standard solution was prepared by diluting the 1.00 mL of standard with 12.5 mL chloroform-methanol (2:1, *v*/*v*) to yield a mixture with a concentration of 0.500 μg/μL for each component. A PI standard solution (No. 1048, Matreya) was prepared at a concentration of 0.500 μg/μL in chloroform-methanol (2:1, *v*/*v*). The two standard solutions were then mixed in an equal volume to create a solution with a concentration of 0.250 μg/μL for each phospholipid. The standard for neutral lipid analysis was Non-Polar Lipid Mixture B (No. 1130, Matreya), containing 25.0 mg total lipid in 1.00 mL of chloroform comprising the following markers: cholesteryl oleate as marker for SE; methyl oleate as marker for ME; triolei as marker for TAG; oleic acid as marker for FFA; and cholesterol as marker for FS. The standard was diluted in a 25 mL volumetric flask with chloroform-methanol (2:1, *v*/*v*) to give a 0.200 μg/μL concentration of each neutral lipid marker. Squalene standard was obtained from Sigma-Aldrich in a 10 mL vial (No. S3626) and diluted in chloroform-methanol (2:1, *v*/*v*) to make a standard solution with a concentration of 0.100 μg/μL.

Analyses were performed on 10 cm *×* 20 cm HPTLC silica gel glass plates with channels and a preadsorbent zone (No. 61927, Miles Scientific, Newark, DE, USA). Before use, plates were prewashed by development to the top with dichloromethane-methanol (1:1, *v*/*v*), dried with a stream of warm air from a hair dryer in a fume hood, and activated for 20 min on a CAMAG plate heater (CAMAG Scientific Inc., Wilmington, NC, USA) at 120 °C.

Standard and reconstituted sample solutions were applied in 2.00, 4.00, 8.00, and 16.0 μL aliquots (0.400 to 3.20 μg standard for neutral lipids, 0.200 to 1.60 μg for squalene, and 0.500 to 4.00 μg for phospholipids) to the HPTLC plates using a 10 μL Drummond digital micropipet. This method using four concentrations of standard was previously validated in Hunsberger et al. [26]. For phospholipid analysis, the Wagner mobile phase was used: chloroform-methanol-water (65:25:4.5, *v*/*v*/*v*). For neutral lipid analysis, the Mangold mobile phase was used: petroleum ether-diethyl ether-glacial acetic acid (40:10:0.5, *v*/*v*/*v*). To resolve and quantify fast moving neutral lipid bands (squalene, ME, and SE), plates were developed with the Smith mobile phase: hexane-petroleum ether-diethyl ether-glacial acetic acid (50:20:5:1, *v*/*v*/*v*/*v*). The use of these mobile phases in HPTLC was recently reviewed in Bui et al. [24]. One-dimensional ascending development was carried out in an HPTLC twin trough chamber (CAMAG) containing a saturation pad (Miles Scientific). The chamber was equilibrated with the mobile phase vapors for 20 min before HPTLC plate development. Mobile phase was allowed to migrate within 1 cm of the top of the plates, requiring approximately 10–15 min.

After development, plates were dried with a stream of cool air in a fume hood for about 10 min. For phospholipid analysis, HPTLC plates were sprayed with 10% cupric sulfate in 8% phosphoric acid and heated at 140 °C for approximately 30 min to detect brown-black bands on a white background. For neutral lipids, plates were sprayed with 5% phosphomolybdic acid (PMA) in ethanol and heated at 120 °C for approximately 30 min to detect blue bands on a yellow background. Lipid bands were quantified against standards by slit-scanning densitometry in the absorbance-reflectance mode using a CAMAG TLC Scanner 3 with slit dimensions 4.00 mm *×* 0.45 mm Micro and scanning rate 20 mm/s. The deuterium light source was set at 370 nm for phospholipid scanning and the halogen-tungsten lamp at 610 nm for neutral lipids. The winCATS software automatically generated polynomial regression calibration curves (standard zone weights versus peak areas) and interpolated sample zone weights based on their peak areas. The percentage by weight of lipid in each wet sample was calculated using the equation: Percent lipid = 100*wR*/sample weight in μg

(1) Percentlipid=100wR/sampleweightinμg

where *w* is the lipid mass (μg) of sample interpolated from the calibration curve and *R* is the reconstitution volume (μL)/spotted volume (μL). If the area of more than one sample aliquot was bracketed within the calibration curve, the weight of the aliquot giving a scan area closest to the average area of the two middle standards was used for calculations. If the sample areas were out of range of the calibration curve, a dilution or concentration step was done, and the sample was analyzed again with the dilution/concentration factor taken into account.

A single-factor ANOVA test was done to check for significant differences for each lipid class among the [*RNQ*^+^], [*rnq*^−^], and Δ*rnq1* samples. If *p*-value was greater than 0.05, there would be no significant differences in that lipid group, and no further analysis would be done. Otherwise, a paired *t*-test was done between [*RNQ*^+^] vs. [*rnq*^−^], [*rnq*^−^] vs. Δ*rnq1*, and [*RNQ*^+^] vs. Δ*rnq1* to determine which pair showed significant difference in a specific lipid class (*p* < 0.05).

## 3. Results

**Figure 1:**
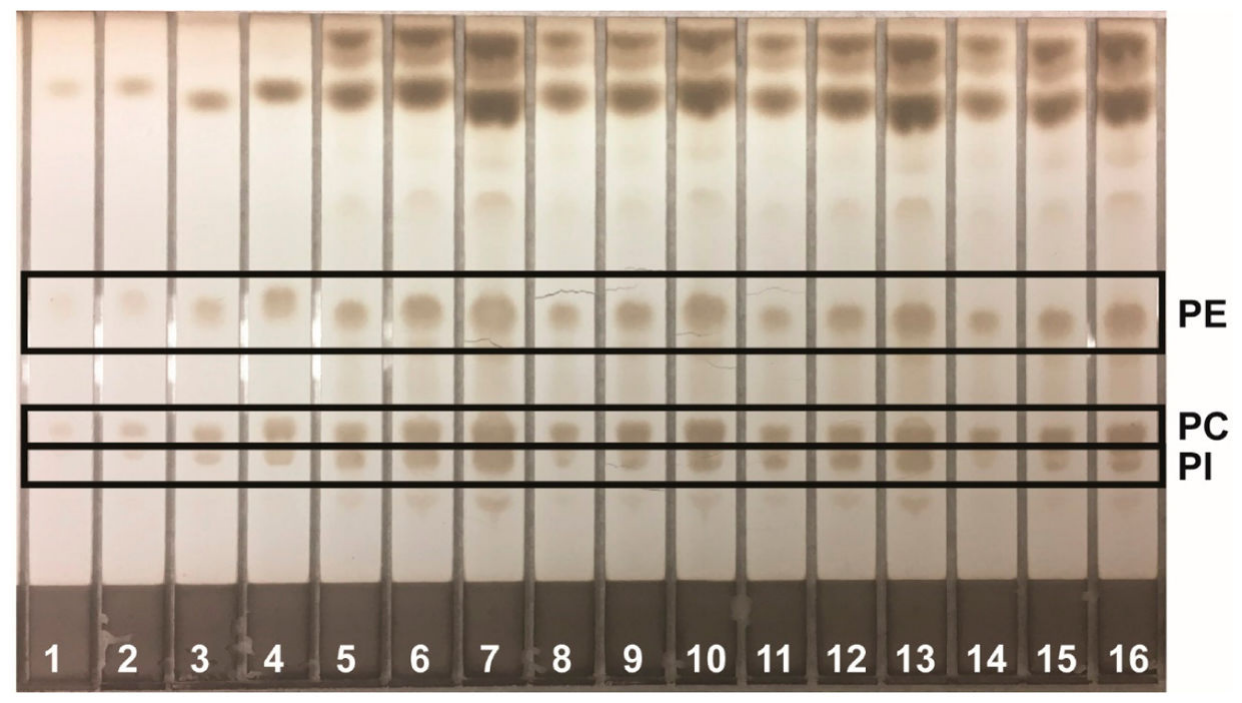
Example of HPTLC plate for analysis of phospholipids using the Wagner mobile phase. Lanes 1–4 are mixed phospholipid standard solution applied in 2.00, 4.00, 8.00, and 16.0 μL aliquots, respectively. Lanes 5–7, 8–10, 11–13, and 14–16 are four typical reconstituted sample solutions applied, respectively, in 4.00, 8.00, and 16.0 μL aliquots each.

**Figure 2:**
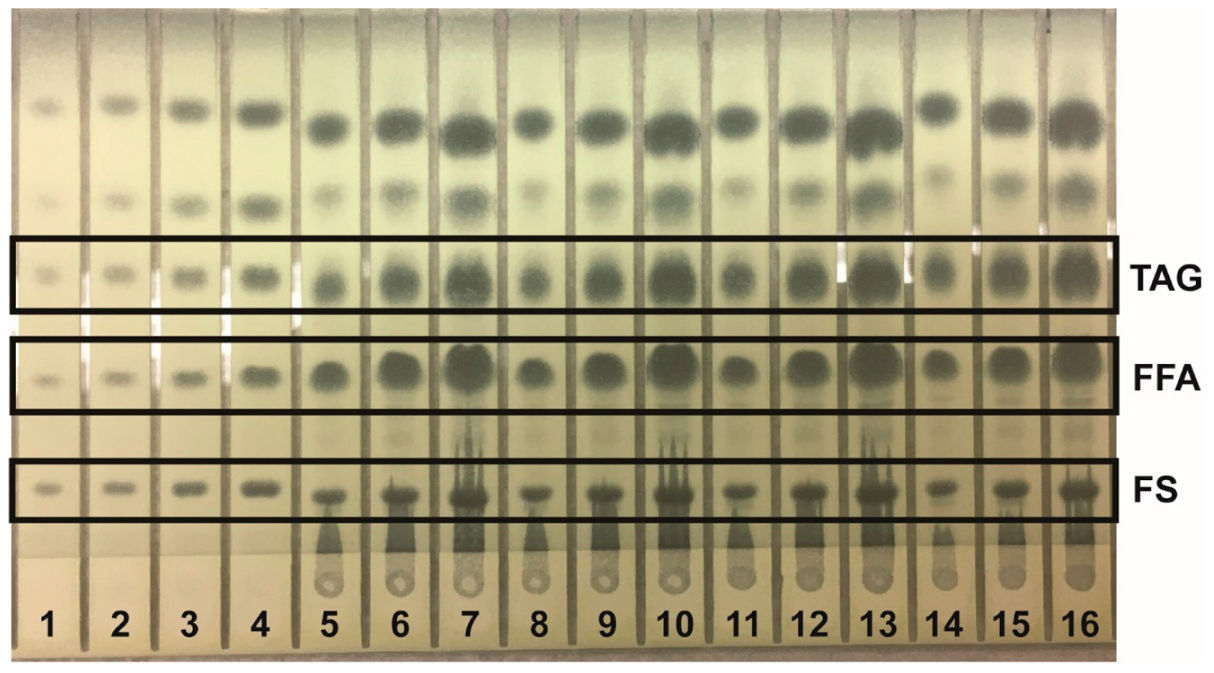
Example of HPTLC plate for analysis of neutral lipids using the Mangold mobile phase. Lanes 1–4 are mixed neutral lipid standard solution applied in 2.00, 4.00, 8.00, and 16.0 μL aliquots, respectively. Lanes 5–7, 8–10, 11–13, and 14–16 are four typical reconstituted sample solutions applied, respectively, in 4.00, 8.00, and 16.0 μL aliquots each.

**Figure 3:**
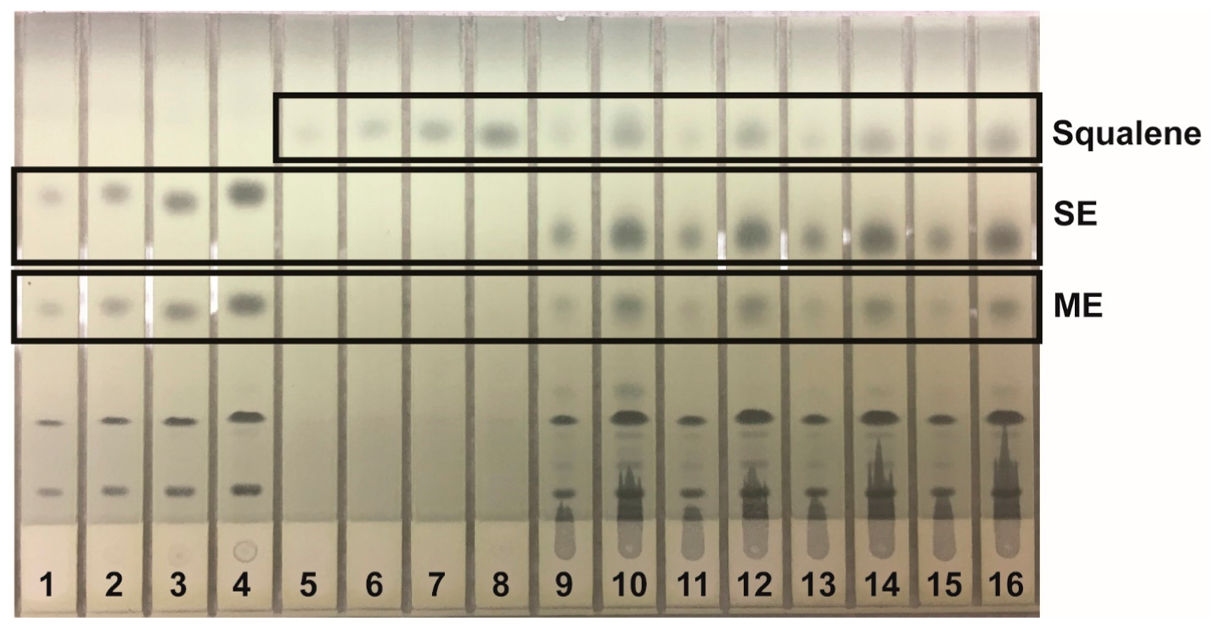
Example of HPTLC plate for analysis of neutral lipids using the Smith mobile phase. Lanes 1–4 are mixed neutral lipid standard solution applied in 2.00, 4.00, 8.00, and 16.0 μL aliquots, respectively. Lanes 5–8 are squalene standard solution applied in 2.00, 4.00, 8.00, and 16.0 μL aliquots, respectively. Lanes 9–10, 11–12, 13–14, and 15–16 are four typical reconstituted sample solutions applied, respectively, in 2.00 and 8.00 μL aliquots each.

Lipids were identified by agreement between the *R*_f_ values of standard and sample zones in each mobile phase. In the Mangold mobile phase, the *R*_f_ values of the standards were cholesterol, 0.10; oleic acid, 0.33; and triolein, 0.51. In the Smith mobile phase, the *R*_f_ values of standards were methyl oleate, 0.41; cholesteryl oleate, 0.56; and squalene, 0.77. In the Wagner mobile phase, the *R*_f_ values of standards were PI, 0.21; PE, 0.27; and PC, 0.48. The Scanner 3 automatically carried out polynomial regression to create calibration curves from standard zones with regression coefficients of at least 0.99 for each analyte and interpolated weights of bracketed sample zones. Standard and sample chromatograms showed good separation between analytes; examples are shown as follows: analysis of phospholipids using the Wagner mobile phase (Figure 1); analysis of neutral lipids using the Mangold mobile phase (Figure 2); and analysis of neutral lipids using the Smith mobile phase (Figure 3).

The results of lipid analysis are presented in Table 1 as percent by weight with respect to initial yeast pellet wet weights. From the comparison between the lipid profiles of the Δ*rnq1* and [*RNQ*^+^] strains against the wild-type ([*rnq*^−^]) strain (Table 2), both the Δ*rnq1* and [*RNQ*^+^] strains displayed more deviations from the wild-type lipid profile when grown in stationary phase compared to logarithmic phase. In the logarithmic phase, the Δ*rnq1* strain showed significant differences in squalene, ME, and SE lipid classes; the [*RNQ*^+^] strain showed significant differences in levels of TAG and squalene, whereas in the stationary phase the Δ*rnq1* strain displayed differences in all lipid classes except for squalene, ME, and PE, and the [*RNQ*^+^] strain showed significant differences in all lipid classes except for TAG, ME, and PE. Furthermore, for stationary phase cultures, the trend in which each lipid class deviated from the wild-type lipid profile was very similar for both the Δ*rnq1* and [*RNQ*^+^] profiles. In fact, the only differences in trend between the Δ*rnq1* and [*RNQ*^+^] profiles when comparing to the wild-type strain were in the TAG and squalene. While both strains showed similar decrease in TAG and increase in squalene levels, only the changes in TAG of the Δ*rnq1* strain and squalene of the [*RNQ*^+^] strain were significant.

The comparison between the lipid profiles of the Δr*nq1* and [*RNQ*^+^] strains, however, showed a reverse trend (Table 3). Most differences between Δr*nq1* and [*RNQ*^+^] were observed during the logarithmic growth phase rather than the stationary phase. In the logarithmic phase, significant differences were observed in all lipids except for FS and PI, whereas, in the stationary phase, the only significant differences observed were in FS, TAG, and squalene. These differences between the Δ*rnq1* and [*RNQ*^+^] profiles did not show any unified trend.

## 4. Discussion

When proteins aggregate to form prions, they typically lose all or most of their normal functionality. Concordantly, we expected to see similar effects (relative to the wild-type [*rnq*^−^] strain) of both the presence of the [*RNQ*^+^] prion and the loss of the Rnq1 protein (Δ*rnq1*). Interestingly, that expectation was met in cells in stationary, but not logarithmic, growth phase (Table 2). Regarding stationary phase cells, the loss of the Rnq1 protein caused an overall reduction in level of neutral lipids, except for squalene (although the level of ME was also slightly reduced, the change was not statistically significant). Similar trends were observed in the [*RNQ*^+^] strain. Together, these observations suggest that the loss of the function of the Rnq1 protein results in a reduction of neutral lipids during stationary phase, which may indicate the involvement of Rnq1 in either neutral lipid synthesis or in a regulatory process that affects these molecules during stationary growth.

We also observed differences between the Δ*rnq1* and [*RNQ*^+^] lipid profiles, despite both strains being expected to experience a loss of function of the Rnq1 protein (Table 3). These differences may indicate the effect of the presence of [*RNQ*^+^] prion aggregates themselves. Specifically, while both the Δ*rnq1* and [*RNQ*^+^] strains exhibited reduced levels of FS in stationary phase, the [*RNQ*^+^] strain demonstrated a very high accumulation of squalene (almost three times the level observed in the wild-type strain), whereas the Δ*rnq1* strain had essentially the same amount of squalene as the wild-type strain. This observation, and the fact that squalene is the precursor to sterols synthesis [27], indicate that the presence of [*RNQ*^+^] aggregates may influence either the production or the conversion of squalene to other lipid products.

When comparing the [*RNQ*^+^] strain of our current study to the [*URE3*]/[*RNQ*^+^] strain used in our previous study [18], both strains exhibited a similar significant increase (approximately three-fold) in squalene in stationary phase with respect to the respective prion-free wild-type strains. Both strains also exhibited a reduction in TAG compared to wild-type. Given the results noted above, it seems likely then that the elevated amount of squalene observed in stationary phase in both studies was caused by the presence of [*RNQ*^+^] aggregates specifically, whereas the reduction in TAG may have been caused by loss of Rnq1 function specifically in both strains that possessed the [*RNQ*^+^] prion, however it is impossible to rule out an effect of the [*URE3*] prion from these data alone. In the logarithmic phase, however, we did not observe consistent trends between our two studies. The reason for such difference was not clear. We hypothesize that the combinative effect from the presence of both prions, [*URE3*] and [*RNQ*^+^], may be more significant in logarithmic phase. Subsequent future examinations of strains containing only the [*URE3*] prion or lacking the prion forming protein Ure2 will further resolve the origins of these changes.

Our results again confirmed that prions affect the lipid profile of *S. cerevisiae* cells differently in different growth conditions (logarithmic growth vs. stationary phase, see Table 1 for direct comparisons), a phenomenon that was first observed in our previous study [18]. Four implications of this finding are apparent. First, any studies that aim to find phenotypes related to prion infection should explore more than one growth phase; second, and perhaps more important, any study of lipid content should carefully control for growth phase; third, variations in the growth phase examined in any study may obscure comparisons of lipid profiles between studies; and, fourth, the fact that lipid profile (and therefore cell physiology) can be so dramatically altered by the presence of a prion means that any yeast-based assays examining, for example, drug candidate efficacy should consider the prion status of the cells used as an important parameter in reporting and evaluating the results. The reproduction of any assay in an otherwise genetically identical set of cells, but one harboring a different set of prions, may give dramatically different results, affecting both assay outcomes and future reproducibility.

In summary, our results further demonstrate that HPTLC-densitometry is a powerful tool for revealing subtle changes in the physiology of yeast, including the effects of prion infection. These results underscore the importance of careful control of growth condition and prion status in experimental procedures and provide new clues regarding the physiological effects of [*RNQ*^+^] prion aggregates and potential functions of the Rnq1 protein. Future studies could utilize this technique to examine the effects of a wide variety of genetic and environmental factors on the lipid profile of yeast and other organisms.

## Figures and Tables

**Table 1:** Lipid profiles of all three yeast strains examined in this study. Numbers represent mean percent lipid weight of total sample wet weight ± one standard deviation (*n* = 4 biological replicates for each strain in each growth phase).

Stationary Phase									
	FS	FFA	TAG	Squalene	ME	SE	PC	PE	PI
Δ*rnq1*	0.026 ± 0.002	0.103 ± 0.009	0.108 ± 0.006	0.009 ± 0.001	0.039 ± 0.004	0.11 ± 0.007	0.044 ± 0.005	0.041 ± 0.008	0.06 ± 0.01
[*RNQ*^+^]	0.022 ± 0.003	0.104 ± 0.007	0.15 ± 0.01	0.03 ± 0.005	0.05 ± 0.01	0.13 ± 0.01	0.04 ± 0.01	0.038 ± 0.002	0.071 ± 0.008
wt [*rnq*^−^]	0.05 ± 0.01	0.12 ± 0.01	0.17 ± 0.02	0.0076 ± 0.0008	0.041 ± 0.004	0.16 ± 0.01	0.027 ± 0.002	0.039 ± 0.006	0.10 ± 0.01

**Log phase**									

	FS	FFA	TAG	Squalene	ME	SE	PC	PE	PI
Δ*rnq1*	0.026 ± 0.008	0.041 ± 0.004	0.014 ± 0.001	0.0053 ± 0.0006	0.023 ± 0.001	0.031 ± 0.003	0.04 ± 0.01	0.027 ± 0.008	0.02 ± 0.02
[*RNQ*^+^]	0.03 ± 0.01	0.07 ± 0.02	0.029 ± 0.008	0.018 ± 0.002	0.018 ± 0.003	0.051 ± 0.004	0.065 ± 0.007	0.04 ± 0.01	0.05 ± 0.02
wt [*rnq*^−^]	0.03 ± 0.01	0.05 ± 0.01	0.018 ± 0.004	0.009 ± 0.002	0.018 ± 0.002	0.045 ± 0.007	0.05 ± 0.01	0.0355 ± 0.004	0.034 ± 0.004

**Table 2:** Comparison between the wild-type [*rnq*^−^] strain and either the Δ*rnq1* or the [*RNQ*^+^] strain (percent increase or decrease with respect to wild-type, [*rnq*^−^] strain). Bold text indicates statistically significant changes (*p* < 0.05).

Log Phase									
	Sterol	FFA	TAG	Squalene	ME	SE	PC	PE	PI
Δ*rnq1*	4.4	−23.2	−23.5	−**41.8**	**31.4**	−**32.1**	−22.2	−22.2	−31.4
[*RNQ*^+^]	39.5	31.6	**59.6**	**93.4**	1.2	11.2	23.5	28.7	51.0

**Stationary phase**									

	Sterol	FFA	TAG	Squalene	ME	SE	PC	PE	PI
Δ*rnq1*	−**48.7**	−**16.3**	−**37.0**	18.4	−4.3	−**32.9**	**61.2**	3.9	−**38.7**
[*RNQ*^+^]	−**57.3**	−**15.7**	−12.4	**296.4**	24.1	−**22.7**	**44.7**	−4.7	−**27.3**

**Table 3:** Comparison between the wild-type Δ*rnq1* and the [*RNQ*^+^] strains (percent increase or decrease with respect to the [*RNQ*^+^] strain). Bold text indicates statistically significant changes (*p* < 0.05).

Log Phase									
	FS	FFA	TAG	Squalene	ME	SE	PC	PE	PI
Δ*rnq1*	−25.2	−**41.6**	−**52.1**	−**69.9**	**29.8**	−**38.9**	−**37.0**	−**39.5**	−54.6

**Stationary phase**									

	FS	FFA	TAG	Squalene	ME	SE	PC	PE	PI
Δ*rnq1*	**20.1**	−0.6	−**28.0**	−**70.1**	−22.9	−13.2	11.4	8.9	−15.7
